# The Research Progress of Trastuzumab-Induced Cardiotoxicity in HER-2-Positive Breast Cancer Treatment

**DOI:** 10.3389/fcvm.2021.821663

**Published:** 2022-01-12

**Authors:** Mengmeng Lin, Weiping Xiong, Shiyuan Wang, Yingying Li, Chunying Hou, Chunyu Li, Guohui Li

**Affiliations:** ^1^National Cancer Center/National Clinical Research Center for Cancer/Cancer Hospital, Chinese Academy of Medical Sciences and Peking Union Medical College, Beijing, China; ^2^Department of Cardiology, Shanghai Putuo District Liqun Hospital, Shanghai, China

**Keywords:** trastuzumab, cardiotoxicity, breast cancer, adverse reaction, rational drug use

## Abstract

In recent years, the incidence of breast cancer has been increasing on an annual basis. Human epidermal growth factor receptor-2 (HER-2) is overexpressed in 15-20% human breast cancers, which is associated with poor prognosis and a high recurrence rate. Trastuzumab is the first humanized monoclonal antibody against HER-2. The most significant adverse effect of trastuzumab is cardiotoxicity, which has become an important factor in limiting the safe use of the drug. Unfortunately, the mechanism causing this cardiotoxicity is still not completely understood, and the use of preventive interventions remains controversial. This article focuses on trastuzumab-induced cardiotoxicity, reviewing the clinical application, potential cardiotoxicity, mechanism and discussing the potential interventions through summarizing related researches over the past tens of years.

## Introduction

Currently, the incidence of breast cancer has been increasing year by year, and now has the greatest incidence of malignant tumors worldwide, with obvious geographical differences. According to GLOBOCAN 2020 estimates of cancer incidence and mortality produced by the International Agency for Research on Cancer, female breast cancer has surpassed lung cancer as the most commonly diagnosed cancer, with an estimated 2.3 million new cases (1). Human epidermal growth factor receptor-2 (HER-2) is an important biomarker for breast cancer as well as a therapeutic target. Of breast cancer patients, 15-20% are HER-2 positive, which is usually considered the most serious subtype due to its poor prognosis and high recurrence rate (2, 3). Trastuzumab is a humanized monoclonal antibody directed against HER-2, initially approved as first-line treatment of HER-2-positive recurrent metastatic breast cancer in 1998. Introduction of trastuzumab to chemotherapeutic regimes has significantly increasing the life expectancy of patients with HER-2 positive, aggressive breast cancer. Meanwhile, there have been increasing reports of trastuzumab-induced cardiotoxicity (TIC) in recent years. To date, the most relevant clinical solution for TIC is trastuzumab interruption, but this approach may cause cancer recurrence. Therefore, understanding the mechanism of TIC and the related preventive measures is paramount for the safe and effective treatment of HER2-positive breast cancer patients. Here, we have attempted to provide an overview of our current knowledge of this effect, focusing primarily on clinical manifestations, influencing factors and mechanism. We also discussed the prevention and pretreatment, with the goal of providing reference for related research and clinical use.

## Overview of Trastuzumab for Her-2-Positive Breast Cancer

Trastuzumab is an important HER-2 targeted drug. The gene encoding HER-2 is localized on chromosome 17 (4) and encodes a transmembrane glycoprotein with tyrosine kinase activity that plays an important role in cell survival, proliferation, and differentiation (5, 6). HER-2 is a member of the epidermal growth factor receptor (EGFR) family and has two forms of activation, homodimerization and heterodimerization with other receptors in the family (HER-1, HER-3, HER-4), either of which triggers cellular pathways including MEK/Erk, PI3K/Akt (7, 8). The mechanism of trastuzumab has not been fully elucidated and may be related to inhibiting the formation of the homodimer by binding to the HER2 extracellular structural domain IV, blocking downstream cellular pathways and thus blocking tumor cell proliferation (9, 10). Recently, Tsao et al. (11) found that the dominant therapeutic mechanism of trastuzumab is through its elicitation of tumor associated macrophages, which mediated antibody-dependent cellular phagocytosis. After HER2 overexpression was discovered to be associated to poor clinical outcomes in breast cancer patients, it quickly became the focus of intensive investigations. In 1989, Hudziak et al. (12) found that a mouse monoclonal antibody to HER-2 successfully inhibited the proliferation of breast cancer cells. Researchers humanized mouse-derived 4D5 monoclonal antibodies and the most active of these was named trastuzumab (13). It was approved by the FDA in 1998 for first-line treatment of HER-2-positive recurrent metastatic breast cancer. Trastuzumab in combination with other agents significantly prolonged median survival (25.1 vs. 20.3 months; *p* < 0.008), progression-free survival (7.4 vs. 4.6 months; *p* < 0.001), improved objective remission rates (50 vs. 32%; *p* < 0.001), and reduced one-year mortality (22 vs. 33%; *p* < 0.008) (14). Several large foreign clinical trials have shown that the use of trastuzumab after receiving chemotherapy can significantly reduce the risk of breast cancer recurrence and death (15–17). Furthermore, a joint analysis of two large clinical trials (NCCTG N9831 and NSABP B-31) found that patients with early-stage HER2-positive breast cancer benefited from the addition of trastuzumab to conventional chemotherapy followed by treatment with paclitaxel, resulting in a significant and sustained reduction in cancer recurrence rates and a 37% improvement in overall survival (18). Both *Chinese guidelines for diagnosis and treatment of pancreatic cancer 2019* and *NCCN Clinical Practice Guidelines in Oncology* recommend trastuzumab as the first choice in combination with chemotherapy drugs (19).

## Clinical Manifestations of Trastuzumab-Induced Cardiotoxicity

It is generally accepted that, unlike anthracyclines, the cardiotoxicity caused by trastuzumab is not dose-dependent, does not occur in all patients, and is reversible (20). Left ventricular dysfunction (LVD) and heart failure (HF) are relatively common and severe manifestations of cardiotoxicity in cancer therapy (21). The Cardiac Review and Evaluation Committee (CREC) defined the cardiotoxicity as one of the following: (1) cardiomyopathy characterized by a decrease in cardiac left ventricular ejection fraction (LVEF) that was either global or more severe in the septum; (2) symptoms of congestive heart failure (CHF); (3) associated signs of CHF, including but not limited to S3 gallop, tachycardia or both; and (4) decline in LVEF of at least 5 to <55% with accompanying signs or symptoms of CHF or a decline in LVEF of at least 10% to <55% without accompanying signs or symptoms (22). Any of the above can be defined as cardiotoxicity. A frequently used definition of treatment-related cardiotoxicity in clinical trials is an absolute decrease in LVEF of 10% to a value of <55% (23). Of these definitions, there may be differences between individual patients regarding the decrease in LVEF. Researchers analyzed 1,437 echocardiograms from 324 patients over a follow-up period of up to 3.5 years, and revealed three main patterns of LVEF change over time: (1) steady decline over time; (2) mild early and late sustained decline; (3) early significant decline with late partial recovery (24).

In addition to left ventricular dysfunction and heart failure, studies also reported the development of arrhythmias, sick sinus node syndrome, and atrial flutter in patients undergoing treatment with trastuzumab (25). Recently, through a secondary analysis of a clinical trial, investigators found that TIC is characterized by the presence of both left ventricular dysfunction and reversible myocardial inflammation and edema, and that trastuzumab may be associated with deleterious changes in cardiac metabolic phenotype (26).

## The Incidence and Influencing Factors of TIC

Many clinical studies have demonstrated the cardiotoxicity associated with trastuzumab, and this article focuses on a few large clinical studies of adjuvant therapy with combination or sequential trastuzumab. In the N9831 study (27), in the two trial groups using trastuzumab, the cumulative incidence of CHF or cardiac death over 6 years was 2.8 and 3.4%, respectively, resulting in risks that were 4.7 and 5.7 times higher than not using trastuzumab. The BCIRG006 (17) study found that the addition of trastuzumab after anthracycline treatment significantly increased the odds of CHF, and the risk of decreased LVEF was 1.6 times greater than without trastuzumab. Furthermore, the incidence of cardiac events reported by NSABP B-31were 1.3% in the control group and 4.0% in the trastuzumab group, with 15.5% of the trastuzumab group discontinuing the drug for cardiac reasons (28). The BIG1-01 (HERA) study (16, 29) conducted a comparative trial of 5102 HER-2 positive early-stage breast cancer patients over 1 and 2 years, respectively. Although the incidence of severe CHF was 0.8% in both groups, the incidence of asymptomatic drop in LVEF was significantly higher in patients on trastuzumab for 2 years (7.2%) than for 1 year (4.1%). The rate of discontinuation of treatment due to TIC was 5.2% during the 1-year period and 9.4% during the 2-year period. Additionally, an 11-year follow-up of the study found that the most of the TIC occurred during the patients' dosing period and no delayed cardiotoxicity was seen (See Table 1).

**Table 1:** Cardiac toxicity induced by trastuzumab.

**TRIAL (Ref.)**	**Median follow-up time**	**Enrolled patients**	**Design**	**Asymptomatic drop in LVEF (≥10-55%)**	**Severe CHF/CE**
NCCTG(Alliance)N9831 (27)	9.2	1,944	AC-paclitaxel AC-paclitaxel-H AC-paclitaxel plus H-H	20.5% 19.6% 22.5%	0.6% 2.8% 3.4%
BCIRG006 (17)	5	3,222	AC-docetaxel plus H AC-docetaxel Docetaxel-carboplatin-H	18.6% 11.2% 9.4%	2.0% 0.7% 0.4%
NSABP B-31 (28)	7	1,830	AC-paclitaxel AC-paclitaxel plus H-H	NO MENTIONED	1.3% 4.0%
HERA(BIG1-01) (29)	8	3,387	Observation 1 Year of H 2 Year of H	0.9% 4.1% 7.2%	0 0.8% 0.8%
PHARE (30)	3.5	3,384	1 year of AC-H 6 months of AC-H	6%(CHF, or LVEF ≥ 10-55%) 2%(CHF, or LVEF ≥ 10-55%)	

*A, anthracyclines; C, cyclophosphamide; H, trastuzumab; CE, cardiac event.*

*CHF, congestive heart failure*.

The incidence varies depending on the assay and criteria for cardiotoxicity used by researchers amongst the different clinical trials, as well as on the selection of patients participating in the trials. For example, in the HERA trial, a lower incidence of cardiotoxicity may be due to the exclusion of patients who had a cardiac event prior to treatment from the trial. Because patients with significant disease, including those at high risk for cardiovascular disease, are often excluded from randomized controlled trials, the incidence may differ from the real world. A real-world study based on trastuzumab for cardiotoxicity due to HER2-positive breast cancer that included more than 3,700 study subjects showed a CHF incidence of 2.8%, with a 1.0% incidence of severe CHF (31).

Risk factors for development of TIC include previous anthracycline exposure and conventional cardiovascular risk factors. Several clinical studies have demonstrated that previous anthracycline exposure appears to be the most important factor in worsening cardiotoxicity (32, 33). This may be related to the fact that the inhibition of the HER2 pathway by trastuzumab exacerbates damage caused by oxidative stress induced by anthracyclines, allowing for further accumulation of ROS (34).

In addition to co-administration, conventional cardiovascular risk factors have been associated with TIC. A recently published systematic review and meta-analysis focusing on the relationship between conventional cardiac risk factors and trastuzumab-induced cardiotoxicity in breast cancer treatment showed that age ≥ 60 (OR 2.03, 95% CI 1.38-3.00, *P* = 0.0004), hypertension (OR 2.01, 95% CI 1.30-3.09, *P* = 0.002), smoking (OR 1.33, 95% CI 1.07-1.65, *P* = 0.01), diabetes (OR 1.49, 95% CI 1.22-1.81, *P* = 0.0001), family history of coronary artery disease (OR 5.51, 95% CI 1.76-17.25, *P* = 0.00001), known history of coronary artery disease (OR 6.27, 95% CI 2.22-17.69, *P* = 0.0005) were strongly associated with the development of TIC (35). Besides, combination of obesity and being overweight was also a significant influencing factor (36).

## Mechanism of Trastuzumab-Induced Cardiotoxicity

The exact mechanism of TIC has not been fully elucidated, and numerous *in vitro* and *in vivo* studies suggest that it may involve multiple cellular and molecular mechanisms (37). The inhibition of NRG-1/HER and downstream signaling pathways has always posed a plausible explanation for TIC, but the underlying molecular mechanisms still remain undefined. In addition, recent research has investigated the inhibition of autophagy and alterations in cellular metabolic pathways in cardiomyocytes as potential causes for the development of cardiotoxicity.

### Downregulation of HER2 Signaling and Cardiotoxicity

In addition to being expressed in tumor tissue, HER2 has been shown to be expressed in adult cardiomyocytes along with other members of the family (HER1, HER3 and HER4) (8). HER2, together with its ligand, NRG1, is closely tied to the maintenance of adult cardiac function and the development of cardiomyocytes. When the heart becomes hemodynamically unstable or stimulated, cardiac microvascular endothelial cells can release NRG1 (38, 39). After acting in a paracrine form in cardiomyocytes, NRG1 binds to HER4 and triggers HER4/HER4 homodimerization or HER4/HER2 heterodimerization, which can later trigger a series of pathways including the MAPK pathway and PI3K-Akt (40).

The activation of the Akt family can trigger many proteins through phosphorylation, thereby initiating tumor cell survival and inhibiting apoptosis (41). Ravingerova et al. (42) used a chronic cardiac ischemia rat model to discover that Akt also increases glucose and lipid metabolism in cardiomyocytes through nutrient uptake and ensures energy in cardiomyocytes during hypoxia. Furthermore, the activation of the PI3K-Akt pathway promotes nitric oxide (NO) production in adult ventricular myocytes, thereby protecting them from oxidative stress. Moreover, Akt can initiate alterations in mitochondrial respiration, thereby reducing reactive oxygen species (ROS) production and improving cell survival. If HER2 signaling is blocked, PI3K-Akt pathway blockade will cause the accumulation of ROS in cardiomyocytes, thereby triggering the apoptosis of cardiomyocytes (43).

The MAPK pathway is another pathway associated with TIC. The MAPK pathway consists mainly of three protein kinases, Raf/MEK/ERK, that cascade to amplify external signals and thus cause cell proliferation and differentiation (44). Meanwhile, the phosphorylation of ERK1/2, inhibits the opening of the mitochondrial osmotic transition pore and suppresses the decrease in membrane potential, thus stabilizing mitochondrial function (45).

**Figure 1:**
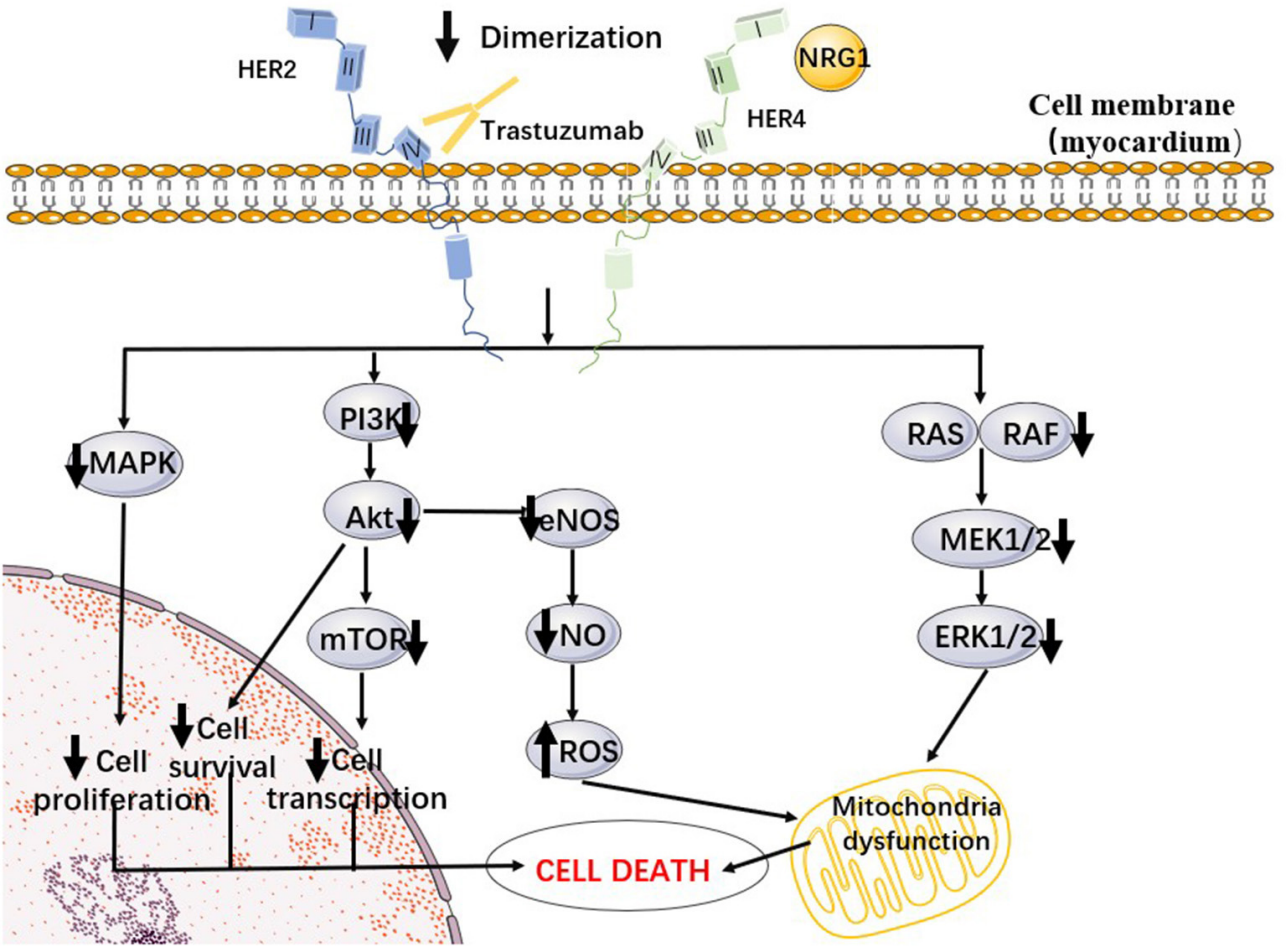
A proposed cellular mechanism of the cardiotoxicity of trastuzumab. Trastuzumab inhibited Her2/4 dimerization, preventing autophosphorylation and subsequent downstream pathways such as PI3K/Akt and MAPK.

In summary, the activation of NRG1/HER and downstream signaling pathways plays an important role in protecting the stability of cardiac function. Trastuzumab inhibits the dimerization of HER4/HER2 by binding to HER2 and thereby inhibiting the above pathways (see Figure 1), which may be one of the potential mechanisms for TIC. In fact, NRG1/HER signaling in the heart is part of a stress-activated compensatory system that plays a minor role under physiological conditions, but can play a protective role when the heart is exposed to cardiotoxic drugs or ischemia, which is consistent with the reality that trastuzumab increases cardiotoxicity when combined with anthracyclines (46, 47).

### Inhibition of Autophagy

Autophagy is a catabolic process that aims to recycle cellular components and damaged organelles in response to different stress conditions (48). Thomas et al. found that deletion of the anti-apoptotic protein MCL-1 in mouse cardiomyocytes leads to the inhibition of autophagy, eventually resulting in heart failure, and further indicated that MCL-1 deficiency is associated with mitochondrial dysfunction (49). Mohan et al. found that trastuzumab treatment decreased the protein expression of autophagy-related signaling molecules such as ATG5-12, ATG7, ATG14, and Beclin 1, and also demonstrated that trastuzumab-mediated inhibition of autophagy resulted in increased ROS production in cardiomyocytes (50). In earlier years, some researchers found that anthracycline increases autophagy and that this is closely related to its cardiotoxicity, which also suggests that anthracyclines and trastuzumab differ in their mechanisms of inducing cardiotoxicity (51, 52).

### Alterations of Cardiomyocyte Metabolism

The inhibition of the NRG1/HER signaling pathway still does not fully answer the question of why trastuzumab causes cardiotoxicity. For example, TIC is often reversible in clinical settings, which contradicts the above explanation that blocking the HER2 pathway leads to cardiomyocyte apoptosis. Alterations in cardiac energy metabolism are a key feature of heart failure and are thought to exacerbate its progression (53). Necela et al. (54) found that after trastuzumab treatment, there was a reduction in glucose uptake in human induced pluripotent stem cell-derived cardiomyocytes (IPSC-CMs) as well as a significant downregulation of SLC6A6. SLC6A6 is a metabolism-related gene, and SLC6A6 knockout mice exhibit a cardiomyopathy with myocardial atrophy phenotype, which also provides a potential mechanism for TIC (55). Recently, investigators have found that clinically relevant doses of trastuzumab impaired the contractile and calcium regulatory functions of IPSC-CMs but did not lead to cardiomyocyte death, and that further RNA-SEQ with subsequent functional analysis revealed that mitochondrial dysfunction and altered cardiac energy metabolic pathways were the main causes of the TIC phenotypes, thus suggesting that metabolic modulators are important for the treatment of TIC (56).

## Prevention and Treatment of TIC

### Monitoring of TIC

Strict monitoring of cardiotoxicity during the treatment of trastuzumab facilitates timely adjustment of dosing and optimization of treatment regimens by clinicians. LVEF, measured by cardiovascular magnetic resonance (CMR) or 2-dimensional echocardiography (2DE), is currently the most commonly used index for monitoring left ventricular function, but LVEF has limitations and often underestimates cardiac compromise in patients. In a retrospective study, investigators found that baseline left ventricular end-diastolic volume (LEVD) was an independent predictor of cardiotoxicity and more reliably identified patients at high risk of cardiotoxicity (57). Besides, echocardiographic measurement of longitudinal shortening of the heart during contraction, or global longitudinal strain (GLS), can identify early changes in left ventricular contractility before ejection fraction (EF) declines. Researchers found thatΔGLS at 6 months were predictors of decrease in EF at 12 months (58). And GLS-guided cardioprotective therapy (CPT) prevents reduction in LVEF and development of cardiac dysfunction in high-risk patients undergoing potentially cardiotoxic chemotherapy, compared with usual care (59). Improvements in testing technology have allowed for the emergence of serum biomarkers that play an increasing role in the monitoring of cardiotoxicity. The 2016 ESC Position Paper on cancer treatments and cardiovascular toxicity published by the European Society of Cardiology (ESC) proposed that the use of serum biomarkers is an important tool for baseline risk assessment and diagnosis of cardiovascular disease. The statement recommends cardiac troponin (CTn) baseline measurement for all cancer patients, as the strongest independent predictor of cardiotoxicity, and in patients with early invasive HER2+ breast cancer undergoing neoadjuvant or adjuvant therapy. B-type natriuretic peptide (BNP)/amino-terminal pro-B-type natriuretic peptide (NT-proBNP) with CTn testing were recommended after receiving trastuzumab (60). In recent years, soluble growth-stimulating expression gene 2 protein (sST2) has received wide attention as a novel heart failure marker. Some studies have shown that sST2 levels correlate with the severity of heart failure, LVEF and NT-proBNP in patients (61). In addition, Zhang et al. (62) analyzed 65 HER2-positive breast cancer patients treated with trastuzumab and applied ordered logistic regression to analyze the relationship between serum miR-222-3p and adverse events and found that serum miR-222-3p was a potential predictor of TIC.

### Choosing an Anthracycline-Free Regimen

In the BCIRG006 clinical trial, the regimen combining anthracyclines with trastuzumab had similar long-term survival rates as the paclitaxel and cyclophosphamide combined with trastuzumab regimen, while the incidence of cardiotoxicity in the latter group was much lower than former (17). A randomized multicenter phase III trial of 438 patients with stage II and III HER2-positive breast cancer showed an estimated 3-year event-free survival rate of 93% in patients treated with anthracyclines and 94% in patients not using, while decrease in LVEF was more common in the anthracycline group (63). This suggests that avoiding anthracyclines when using trastuzumab in favor of other classes of drugs may reduce the likelihood of cardiac events without compromising efficacy.

**Figure 2:**
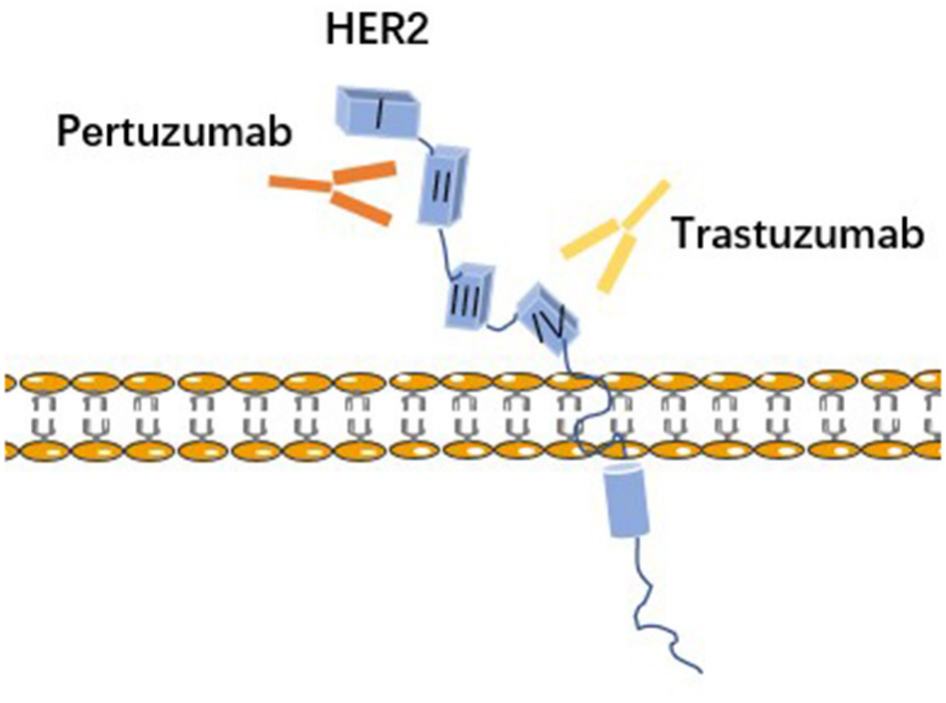
Trastuzumab and pertuzumab bind to different regions on HER2. Trastuzumab is a humanized monoclonal antibody to IV subdomain of HER2. Pertuzumab is a humanized monoclonal antibody to subdomain II of the dimerization arm of HER2.

In addition to its use in combination with chemotherapeutic agents, trastuzumab has shown a good prognosis in combination with other antitumor drugs. Unlike trastuzumab, pertuzumab is a humanized monoclonal antibody against the extracellular structural domain II region of HER2, which inhibits the heterodimerization of HER2 with HER3, thereby blocking pathways including phosphatidylinositol 3-kinase (PI3K/AKT/mTOR) and mitogen-activated protein kinase (RAS/RAF/MEK/ERK) (64, 65). It acts at a different site from trastuzumab in the extracellular structural domain of HER2 and there may be a synergistic effect when they are combined (see Figure 2). The NeoSphere phase II study evaluated the efficacy and safety of trastuzumab with pertuzumab in combination with docetaxel in HER2-positive breast cancer patients treated with neoadjuvant therapy, and showed that the dual-target combination chemotherapy significantly increased the pathologic complete remission rate (pCR) as compared to the single-target, while the adverse effects were broadly consistent with the trastuzumab monotherapy arm (66). Furthermore, the TRYPHAENA trial demonstrated that the combination of trastuzumab and pertuzumab, whether co-administered with anthracyclines or with carboplatin, was usually well-tolerated and also showed a higher rate of pCR and a lower incidence of cardiotoxicity in the anthracycline-free trial group (67). Similarly, the PEONY study demonstrated that dual-targeted combination therapy significantly improved the pCR rate (68). The 2019 NCCN guidelines recommend the TCHP regimen [trastuzumab (H) + pertuzumab (P) in combination with docetaxel (T) + carboplatin (C)] as a first-line treatment option for HER-2-positive breast cancer. This regimen is anthracycline-free and therefore has a higher safety profile for patients with potentially dangerous cardiac function.

### Pharmacological Prevention

Unlike anthracyclines, LVD caused by trastuzumab is usually reversible, thus the ESMO guidelines mainly recommend strategies such as observation and discontinuation of the drug (69). However, a large retrospective cohort study found that discontinuation of trastuzumab led to adverse clinical outcomes (70). Therefore, it is necessary to use appropriate cardioprotective agents in the clinical setting.

#### Angiotensin-Converting Enzyme Inhibitor or β-Receptor Inhibitors

Early research found that NRG-1/HER signaling regulates myocardial contractility and is influenced by circulating catecholamines and angiotensin-II in animal models (39). This may provide some theoretical basis for the use of ACEI or β-receptor inhibitors in the prevention of TIC. Several small randomized trials and single-center studies have also reported that ACEI and β-blockers ameliorate chemotherapy-induced cardiotoxicity, but these studies all emphasized high-dose anthracycline-induced cardiotoxicity and have limited clinical applicability in TIC (71, 72). In 2016, the MANTICORE 101-Breast randomized clinical trial specifically investigated the pharmacological prevention of TIC and found that Perindopril and Bisoprolol were well-tolerated in the prevention of TIC and attenuated the decrease in LVEF, but trastuzumab-induced left ventricular remodeling was not reversed (73). In another randomized clinical trial, however, concomitant use of the angiotensin II receptor inhibitor Candesartan did not prevent a reduction in LVEF (74). In 2019, researchers evaluated the preventive effect of Lisinopril and Carvedilol on cardiotoxicity with or without anthracyclines prior to trastuzumab administration and found that both drugs were more protective in patients who had exposure to anthracyclines. Patients receiving pharmacological interventions were more likely to benefit compared to the placebo group [(75); Table 2].

**Table 2:** Primary cardiac prevention studies in patients with breast cancer receiving trastuzumab.

**References**	**Enrolled patients**	**Treatment**	**Cardiac prevention strategy**	**Results**
Heck et al. (72)	130	Epirubicin (*n* = 28 with trastuzumab)	candesartan 32 mg, metoprolol 100 mg, placebo (2 x 2 design)	Absolute LVEF change: 2.6% in placebo, 0.8% in candesartan;
Pituskin et al. (73)	99	Trastuzumab (*n* = 22 with doxorubicin)	perindopril 8 mg, bisoprolol 10 mg, placebo (1:1:1)	LVEDVI not different among arms
Boekhout et al. (74)	206	Epirubicin with trastuzumab	candesartan 32 mg, placebo	LVEF decline: 19% in candesartan, 16% in placebo
Guglin et al. (75)	468	Trastuzumab (*n* = 189 with doxorubicin)	carvedilol 10 mg, lisinopril 10 mg, placebo	LVEF decline: 32% in placebo, 29% in carvedilol, 30% in lisinopril.

*LVEDVI, left ventricular end-diastolic volume index*.

#### Statins

A retrospective case control study found that in women with HER2+ breast cancer receiving trastuzumab-based therapy with or without anthracyclines, concomitant statin use was associated with a lower risk of cardiotoxicity (76). And a recent meta-analysis also showed that patients receiving statins during cancer treatment had a lower incidence of cardiotoxicity and were more likely to maintain LVEF during the follow-up period, suggesting that statins have the potential to mitigate the cardiotoxic effects of anthracyclines and trastuzumab (77). Rosuvastatin is a statin with anti-lipid peroxidation effects (78). Kabel et al. (79) found that rosuvastatin had a protective effect against TIC in mice due to the antioxidant and anti-inflammatory properties combined with its ability to induce STAT-3 expression and preserve the morphology of the cardiomyocytes. This study also demonstrated better results in combination with ubiquinone, the oxidized form of coenzyme Q10.

#### AMPK Agonist

AMPK (Adenosine 5'-monophosphate (AMP)-activated protein kinase) is considered to be a key regulatory kinase of myocardial energy metabolism (80). Recently, researchers identified mitochondrial dysfunction and altered cardiac energy metabolic pathways as important potential mechanisms of trastuzumab-induced cardiotoxicity (56). Susheel et al. (81) found that low-dose metformin improved mitochondrial function and provided significant myocardial protection against ischemic heart failure by activating AMPK and downstream signaling pathways involving eNOS and PGC-1. Wang et al. (82) observed a heterodimeric shift of AMPKα2 to AMPKα1 in the hearts of heart failure patients and mice with transverse artery constriction. They further found that overexpression of AMPKα2 prevented drug-induced chronic heart failure by increasing mitochondrial phagocytosis and improving mitochondrial function in isolated adult mouse cardiomyocytes. This is consistent with the finding that AMPK agonists (AICAR, metformin) improve trastuzumab-induced symptoms of cardiac insufficiency in IPSC-CMS (56). Although there are no relevant clinical studies to prove whether an AMPK agonist has the function of preventing trastuzumab cardiotoxicity, targeting cellular energy metabolism is a potential research direction. Additionally, it has been shown that activation of AMPK can inhibit the growth of breast cancer cells and increase the sensitivity of breast cancer as well as various other cancers, to chemotherapy and radiotherapy (83). Therefore, it is of great clinical interest to investigate whether AMPK agonists can be used to combat TIC.

#### Potential Role of Traditional Chinese Medicine on TIC

There are many studies on the prevention and treatment of anthracycline-induced cardiotoxicity in Traditional Chinese Medicine (TCM), but reports regarding TIC are rare. The inhibition of the NRG1/HER pathway is one of the possible mechanisms of TIC. It has been suggested that activation of Akt may protect cardiac function by inhibiting apoptosis (84). Many active ingredients in Chinese medicine have been reported to show protective effects against cardiac injury by interfering with the PI3K/Akt signaling pathway, as shown in Table 3 (85–94). In addition, Zhang et al. (95) used network pharmacology analysis to find that Shenmai injection has multi-target and multi-pathway synergistic effects, which may exert myocardial protective effects through the PI3K-Akt signaling pathway and tumor microRNAs. The Shenmai injection treatment group improved cardiac structure and function, reduced myocardial pathological damage as well as the number of autophagic vesicles in mice compared with the control group. Targeting the inhibition of autophagy by trastuzumab, Liu et al. (96) investigated the protective mechanism of ginsenoside Rg2 against TIC in human cardiac myocytes (HCMs), and found that it could induce autophagy in HCMs by upregulating the expression levels of p-Akt, p-mTOR, Beclin 1, LC3, and ATG5, thus providing protection against TIC. At present, TCM is playing an increasing adjuvant role in the process of cancer treatment, while its role in the prevention and treatment of TIC has yet to be fully explored. Further in-depth studies are of great significance to ensure the safe use of trastuzumab as well as to promote the development of TCM in China.

**Table 3:** Active ingredients of Chinese medicine against cytotoxic drug–induced cardiotoxicity through regulation of the PI3K/Akt signaling pathway.

**Active ingredients of Chinese medicine (Ref.)**	**Experimental model**	**dose/route/time**	**Treatment**	**Findings**
Ferulic acid Apigenin (85)	Wistar rats	100 mg/kg; p.o. for 7 days	Doxorubicin	↓NF-κB/PKC-δ ↓p53/p38/JNK ↑PI3K/ Akt/mTOR
	Cardiomyocytes	50 μM for 24 h		
Salvianolic acid B (86)	BALB/c mice	2 mg/kg; i.v., for 7 days	Doxorubicin arsenic trioxide	↑PI3K/Akt ↓ GSK3β/ER
	Cardiomyocytes	10 μM for 12 h		
Paeonol (87)	BALB/C Mice	50 mg/kg, for 6 days	Epirubicin	↓PI3K/Akt/mTOR ↓NF-κB
	H9c2 cells	50 mg/kg, for 6 days		
Rutin (88)	C57BL/6 mice	30 and 50 mg/kg; i.v. for 7 days	Pirarubicin	↑PI3K/Akt/mTOR ↓NF-κB
	H9c2 cells	10, 30, 50, and 70 μM for 1 h		
Astragalus polysaccharide (89)	C57BL/6 mice	1.5 g/kg; p.o. for 3 days	Doxorubicin	↑PI3K/Akt ↓p38 MAPK
	Rat Cardiac Myocytes	50 μg/ml for 1 h		
Calycosin (90)	Kunming mice	50 and 100 mg/kg; i.p. for 7 days	Doxorubicin	↑PI3K-Akt ↑Sirt1/NLRP3
	H9c2 cells	200 μM for 24h		
Total flavonoids from Clinopodium Chinense (91)	Male Sprague-Dawley (SD) rats	80 mg/kg, i.p. for 15 days	Doxorubicin	↑PI3K/Akt ↑Nrf2/HO-1
	H9c2 cells	6.25, 12.5, 25, and 50 μg/ml for 24 h		
Ginkgolide B (92)	C57BL/10 mice	100 mg/kg, i.p. for 5days	Doxorubicin	↑PI3K/Akt ↓p38 MAPK
	H9c2 cells	1, 5 and 50 μM for 30 min		
Saponins from leaves of Panax Quinquefolius (93)	ICR mice	125 and 250 mg/kg; p.o. for 15 days	Cisplatin	↓NF-κB ↑PI3K/Akt/GSK-3β
Neferine (94)	H9c2 cells	10 μM for 24 h	Doxorubicin	↑IGF-IR/PI3K/Akt

## Summary and Prospects

Trastuzumab is a landmark agent in the treatment of HER2-positive breast cancer. It has changed the treatment paradigm for HER2-positive breast cancer patients and has no alternative to its status as a first-line drug for breast cancer. At the same time, its cardiotoxicity remains a major constraint to its use. The mechanism of trastuzumab cardiotoxicity has not been fully elucidated, and there is no specific drug to prevent it in clinical practice. Fewer studies have been conducted specifically on the cardiotoxicity of trastuzumab than on anthracyclines. Researchers should further clarify the mechanism of TIC, establish a reasonable model of myocardial injury, determine appropriate detection indicators, and conduct research on relevant cardioprotective agents in response to the mechanism in order to provide the possibility of safer use of trastuzumab. In addition, TCM has shown great potential in the prevention of antineoplastic drug-induced cardiotoxicity, and while few studies have been conducted specifically for trastuzumab, this provides a research direction for the prevention and treatment of TIC. There are several hurdles at the clinical study level given that studies evaluating patients treated with trastuzumab alone are lacking, strategies to prevent anthracycline-induced cardiotoxicity are not always applicable to trastuzumab, and the definition and evaluation metrics of cardiotoxicity have yet to be standardized. In clinical application, physicians as well as pharmacists should fully understand the risk factors and fully evaluate basic information such as age, previous cardiovascular history, medication history, and the physical condition of patients before drug administration. In addition, high-risk patients need to be monitored closely throughout the oncology treatment process. These efforts will maximize efficacy while minimizing adverse effects.

## Author Contributions

ML and WX assorted information and drafted the manuscript. SW polished the language. YL and CH offered advice about the structure. CL and GL governed the whole process and offered advice. All authors contributed to the article and approved the submitted version.

## Funding

This work was supported by Beijing Hope Run Special Find of Cancer Foundation of China (LC2020L03) and Beijing Municipal Science and Technology Commission (Z181100001618003).

## Conflict of Interest

The authors declare that the research was conducted in the absence of any commercial or financial relationships that could be construed as a potential conflict of interest.

## Publisher's Note

All claims expressed in this article are solely those of the authors and do not necessarily represent those of their affiliated organizations, or those of the publisher, the editors and the reviewers. Any product that may be evaluated in this article, or claim that may be made by its manufacturer, is not guaranteed or endorsed by the publisher.
